# The introduction of on-scene blood transfusion in a civilian physician-led pre-hospital trauma service

**DOI:** 10.1186/1757-7241-21-S1-S27

**Published:** 2013-05-28

**Authors:** AE Weaver, S Eshelby, J Norton, DJ Lockey

**Affiliations:** 1 London’s Air Ambulance, Royal London Hospital, Whitechapel, London, UK; 2 Barts and the London Medical School, University of London, London, UK

## Background

London’s Air Ambulance (LAA) teams attend over 2000 trauma missions per year. Ten percent of patients suffer from serious haemorrhage. Since 2008, physician-led pre-hospital teams have activated “Code Red – massive haemorrhage protocol” pre-alerts to the Major Trauma Centres in London.[1] A significant number of seriously injured patients die at the scene of the incident. Major haemorrhage contributes to a proportion of these deaths and until recently, fluid resuscitation consisted of crystalloid infusion. Studies have demonstrated dismal outcomes from hypovolaemic traumatic arrest.[2] In March 2012, LAA became the first service in the United Kingdom to routinely carry emergency O Negative blood (packed red blood cells PRBC). Indications to transfuse include traumatic arrest where hypovolaemia is a contributing factor or patients who meet Code Red criteria and require volume resuscitation prior to hospital. Our aim was to examine the impact of on-scene blood transfusion for seriously injured patients.

## Methods

Demographic and clinical data were collected prospectively for patients receiving on-scene blood transfusion, including mechanism of injury, indication for transfusion, on-scene physiology, number of units PRBC transfused on-scene, first in-hospital blood gas values, injuries and outcome data. The volume of blood components transfused, returned to stock and wasted within the first 24 hours was collected retrospectively.

## Results

During six months, 50 patients received on-scene blood transfusion. Mean age 35 years. 40 patients were male (80%). 22 patients suffered traumatic cardiac arrest on scene. Return of spontaneous circulation (ROSC) was achieved in 10 (45%) patients. Mean number of units transfused on-scene was 2.8 units. Mean on scene time was 37 minutes. 39 patients were transported to hospital. One infant was conveyed to hospital in hypovolaemic traumatic cardiac arrest and was pronounced dead in the Emergency Department.

38 patients arrived at hospital with spontaneous circulation. On arrival in the Emergency Department (ED) blood gas values demonstrated mean pH 7.07, mean Base Deficit -12.0, mean haemoglobin 14.0. Eight patients died in ED, 3 patients died in theatre and 4 patients died after admission. Mean values for products transfused in the first 24 hours were 10.5 units PRBC, 8.3 units FFP, 1.6 units platelets, 1.9 units cryoprecipitate.

24 patients survived. 60-day mortality was 52%. 256 units of blood were transfused on-scene. One unit of blood was wasted due to communication failure with the transfusion laboratory when returning an unused unit of blood. 100% traceability was achieved.

## Conclusion

The introduction of on-scene blood transfusion has improved the ROSC rate from traumatic cardiac arrest secondary to hypovolaemia. This has not resulted in a survival benefit in this group of patients. Only one patient did not receive any blood products following arrival at hospital, suggesting that teams can accurately predict the requirement for massive haemorrhage protocols. Blood product ratios appear to be maintained despite on-scene PRBC transfusion. Physician-led pre-hospital teams are able to safely administer on-scene transfusion whilst complying with standard NHS legislation and documentation. Compliance with Standard Operating Procedures ensures that blood products can be returned to hospital supplies if unused with no resultant wastage.
